# 罗特西普联合环孢素A、甲泼尼龙、沙利度胺治疗VEXAS综合征1例

**DOI:** 10.3760/cma.j.cn121090-20250216-00069

**Published:** 2025-07

**Authors:** 洁 周, 南京 王, 丽莲 刘, 葳 李

**Affiliations:** 福建医科大学附属三明第一医院血液内科，三明 365000 Department of Hematology, Sanming First Hospital Affiliated to Fujian Medical University, Sanming 365000, China

患者，男，67岁。2019年5月起出现全身皮疹，表现为结节样丘疹，主要累及颜面、颈部及前胸，伴有瘙痒及疼痛，无畏冷、发热，无头晕、乏力，无胸闷、心悸等。就诊我院风湿免疫科，实验室检查：类风湿因子（RF）19.1 U/ml，红细胞沉降率（ESR）53 mm/1 h，C反应蛋白（CRP）21.83 mg/L；血常规和血生化均正常；ANA和抗ENA试验阴性。左上肢皮肤病理显示鳞状上皮增生，局灶上皮角延长，皮下间质见多量的淋巴细胞浸润（以T淋巴细胞为主），诊断为结节性痒疹。给予卤米松、糠酸莫米松及炉甘石洗剂等外用药物及氯雷他定、苯海拉明等口服抗过敏及甲氨蝶呤免疫抑制治疗。治疗后，皮疹稍消退，瘙痒及疼痛有所缓解，但易反复。2020年8月，患者出现多关节痛，累及双手近端指间关节、双肩、双踝，无明显晨僵，皮疹仍存在。实验室检查：RF 153 U/ml，ESR 142 mm/1 h，CRP 99.9 mg/L，IgG 18 g/L，IgA 5.92 g/L；抗CCP、ANA和抗ENA试验均为阴性，诊断：类风湿性关节炎。予糖皮质激素、甲氨蝶呤、来氟米特、羟氯喹、艾拉莫德、沙利度胺、他克莫司、托法替布和阿达木单抗治疗。关节痛及皮疹仍反复发作，糖皮质激素无法减停。期间症状缓解时曾尝试间断停用激素，但短期内易复发，故长期维持甲泼尼龙（4～8 mg/d）治疗。

2023年11月，患者再次因皮疹、关节痛及乏力外院就诊。血常规示WBC 3.48×10^9^/L，中性粒细胞比值55.2％，HGB 63 g/L，平均红细胞体积（MCV）119.1 fl，PLT 104×10^9^/L。骨髓象：骨髓增生明显活跃，粒红比为2.62∶1；粒系占65.5％，部分细胞胞体大，核质发育不平衡，胞质中颗粒粗大及空泡变性，病态造血细胞占19.0％；红系占25.0％，有核红细胞胞体大，部分细胞有蜂窝状空泡变性，易见多核、畸形核红细胞及H-J小体，病态造血细胞占31.0％，成熟红细胞中心淡染区扩大，呈缗钱样排列；全片见巨核细胞352个，小巨核、单圆巨、多圆巨多见，血小板可见；网状细胞占0.5％，偶见噬血现象。流式细胞术：骨髓标本粒系轻度异常，可见少量异常髓系原始细胞，约占有核细胞的1.92％，主要表达CD33、CD13、CA117、HLA-DR、CD38。骨髓活检：骨髓增生明显活跃，三系可见，粒红比例增高，粒系增生，各阶段粒细胞均可见，幼稚阶段粒细胞增多（MPO^+^、CD34^-^、CD117^-^）；散在红系造血岛，幼稚红细胞增多，晚幼红少（CD235^+^）；巨核细胞增生，呈簇状聚集，部分分叶少（CD61^+^）。间质网状纤维轻度增生（网织染色+）。FISH：CSF1R、EGR1、D7S486、D7S522、D20S108、CSP8缺失探针均阴性。血液系统肿瘤常见285个基因突变检测：UBA1错义突变，ASXL1、IDH1、IDH2、TP53、STAT3、CSF3R、FLT3、TET2等均阴性。诊断：VEXAS综合征；骨髓增生异常综合征；嗜中性皮炎。具体治疗不详，HGB波动在70～82 g/L。

2023年12月，患者因头晕、乏力加剧，伴有全身皮疹和多关节痛，遂就诊我院。查体：体重50 kg，全身皮肤黏膜重度苍白，颜面、四肢及躯干可见暗红色斑疹，双侧关节有压痛和肿胀。于2023年12月13日开始予甲泼尼龙30 mg/d，环孢素A 100 mg每12 h 1次口服及间断输注红细胞支持治疗，治疗过程中皮疹逐渐消退、关节痛明显改善，HGB较前上升。于2024年1月15日起甲泼尼龙、环孢素A逐渐减量，皮疹再起，复查血常规：WBC 3.27×10^9^/L，HGB 60 g/L，PLT 107×10^9^/L；CRP 6.72 mg/L，ESR 108 mm/1 h；遂加沙利度胺50 mg/d抗炎，HGB仍未上升；于2024年3月起加用罗特西普1 mg/kg每3周1次规律治疗5个周期。治疗后关节痛进行性改善，皮肤红色结节样斑丘疹明显减少，HGB进行性改善。此后规律口服环孢素A 75 mg每12 h 1次，甲泼尼龙8 mg/d，沙利度胺50 mg/d维持治疗，维持治疗至2025年1月，药物未减量。共随访7个月，前5个月HGB波动于101～110 g/L，CRP波动于0.91～4.36 mg/L，ESR波动于12～32 mm/1 h，无明显关节痛，活动自如，视觉模拟评分（VAS）0分，皮疹基本消退。第6个月起患者HGB逐渐降至90 g/L，再次加用罗特西普治疗（1 mg/kg每3周1次，共2周期），HGB再次升至116 g/L。

